# Psycopathia Sexualis, with Especial Reference to Contrary Sexual Instincts; a Medico-Legal Study

**Published:** 1893-08

**Authors:** 


PSYCOPATHIA SEXUALIS, WITH ESPECIAL REFERENCE TO CONTRARY SEXUAL INSTINCTS; A MEDICO-LEGAL STUDY, by Dr. R. Van Krafft Ebing, Professor of Psychiatry and Nervous Diseases, University of Vienna.
AN AUTHORIZED TRANSLATION OF THE SEVENTH GERMAN EDITION, by Charles Gilbert Shaddock, M. D., Professor of the Mental Diseases, Marion Sims College, St. Louis. Published by the F. A. Davis Co., Philadelphia and London, 1893.

Having read the original of this translation, it gives me great pleasure to state that Dr. Shaddock has expressed admirably the author's ideas, and the translation is as near the original as possible.

The work is a complete treatise on the subject, and as such stands alone. No other work on the subject so fully deals with the wonderful phases of the pathology of the sexual instructs.

Medico-legal jurisprudence will have an impregnable fortress on which to lean, and suffering humanity will be benefitted in consequence.

Prof. Ebing stands at the head of writers on psychiatry, and America will do him the honor to read the translation of this much needed book. H. H.

